# Annotations

**Published:** 1897-09-11

**Authors:** 


### Annotations.

### The Defects of the Medical Act.

The doctor's charter, the one little bit of protection which he is imagined to receive in return for his long and expensive education, is the fortieth clause of the Medical Act, which prohibits every unqualified person, under a penalty of £20, from wilfully and falsely pretending to be either physician, surgeon, or general practitioner, or taking or using any name, title, addition, or description, implying that he is a registered person, or that he is recognised by law as a physician, a surgeon, a licentiate in medicine and surgery, or a practitioner in medicine, or an apothecary. One would have thought that this clause was ample enough for its purpose, and no doubt those who framed and those who passed the Act did think that it would be effective, and that they were giving to medical men some sort of return for the long and expensive training forced upon them under the Act. No greater mistake, however, could be made. Plain as the matter seems, magistrates have again and again refused to convict, the smallest addition to the usual title, such for example, as the insertion of the word "Homœopathic" before the title "Physician," being accepted as sufficient to alter its meaning, and to exempt it from implying that the user is a registered person. The manner in which prosecutions under this clause are dealt with by the magistrates is a great scandal; not only are medical men, after all their years of study, left exposed to the unrestricted competition of unqualified persons, but the public for whose protection the Act was passed are deprived of the very benefit which it was the chief object of the Act to confer upon them, namely, the power of distinguishing qualified from unqualified practitioners.

### Public Baths and Personal Cleanliness.

Some very admirable public swimming-baths have been erected in London and other large towns, and as a means of providing the people with a most healthy recreation, and giving them an opportunity of learning a most useful and life-saving art, these institutions are worthy of the highest praise. It must be confessed, however, that in many cases everything has not been done that could be done to render public baths attractive as a means of securing personal cleanliness. One of the hardest things that not only the poor, but all who labour at trades of the rougher sort, have to bear, is the necessity of being dirty. What is wanted, however, for their use, is something very different from the ordinary tub-bath. The bath that one has to get into, however refreshing, is by no means an ideal arrangement for cleansing purposes. First of all, it is expensive in consequence of the large quantity of warm water required; secondly, it is ineffectual, the water being soiled at the first touch; lastly, the bath itself becomes dirty and unattractive, if not even dangerous, unless a considerable staff of attendants are employed in following the bathers and cleansing the bath. All these evils are obviated by the spray or rain baths which are so common abroad. If a man can have a warm place in which to undress; if he can then step into a cupboard the bottom of which consists of a foot-bath four inches deep filled with warm water, while by turning a tap he can project upon himself a rain of warm water on every side; if during this process he uses soap; and if when it is over he pulls a string which gives him a final cool shower, he has every requisite for cleanliness, he runs no risk of catching anything from the bath, and the expenditure in water and attendance is reduced to a minimum. A penny, or even a halfpenny, ought in this way to pay for a good wash. These are the sort of baths that are wanted as an aid to cleanliness, not in great establishments, but widely spread and near the people's homes.

### Cosy Houses.

It will be an interesting and by no means an impossible outcome of the visit of our scientific men to Canada if they should return to this country with somewhat enlarged ideas in regard to the best means of warming dwelling-houses. Not that they will have had practical experience, but the very splendour of a Canadian summer must have driven them to inquire as to the means taken to avoid the corresponding rigours of the winter, and if a study of the arrangements there made for house warming should lead to some improvement in those in force in this country many people will have cause to bless this year of Jubilee. The great stumbling-block in the heating arrangements of the ordinary middle-class English house is that attention is concentrated on warming the individual room instead of warming the whole building. The result is that in wandering from room to room the unfortunate inhabitants experience a range of temperature quite as great as between that of the kitchen and the open air. What more icy cavern can be conceived than a "best bed-room," or an unused drawing-room in the winter time? People are sometimes surprised at a medical man declining to take off all his wraps in the hall. He knows better. He knows that while the patient is being "got ready" (hateful custom by which every clue to the progress of the case and the character of the nursing is removed) he will be turned into a chilly drawing-room. If, then, he is wise he keeps on his top coat—if he is wiser still, he turns a deaf ear to the housemaid and walks straight upstairs. What is wanted in all our houses is a heating apparatus in the basement, or, at the least, in the hall, with arrangements for the introduction of fresh air. Draughts are the great bugbear of an English winter. People forget, however, that draughts are only felt because they are thin and because they are cold. A large movement of warm air is hardly felt; but when with a roaring fire, doors and windows are closed "to keep out the draught," thin streams of icy air rushing through every chink and every keyhole swirl about in the warm atmosphere on their way to the fireplace, rendering life unbearable to everyone they touch. If the hall is warm, and if all the staircases are filled with air of a cozy temperature, doors may be opened without fear, and ventilation may easily be effected by large currents instead of by piercing draughts. There is every reason to believe that with a central heating apparatus, properly arranged to allow of the ingress of warm air, the whole of a house could be kept at a comfortable temperature at considerably less cost than is now incurred in keeping a few rooms hot and draughty. Far more colds are caught in chilly passages than in the open air.

